# Assigning the stereochemistry of natural products by machine learning

**DOI:** 10.1186/s13321-026-01205-6

**Published:** 2026-04-25

**Authors:** Markus Orsi, Jean-Louis Reymond

**Affiliations:** Department of Chemistry, Biochemistry and Pharmaceutical Sciences, University of Bern, Freiestrasse 3, 3012 Bern, Switzerland

**Keywords:** Natural products, Chirality, Stereochemistry, Language models, Machine learning

## Abstract

Nature has settled for L-chirality for proteinogenic amino acids and D-chirality for the carbohydrate backbone of nucleotides. Further stereochemical patterns exist among natural products produced by common biosynthetic pathways. Here we asked the question whether these regularities might be sufficiently prevalent among natural products (NPs) such that their stereochemistry could be machine learned and assigned automatically. Indeed, we report that a language model can be trained to assign the stereochemistry of NPs using the open access NP database COCONUT. In detail, our language model, called **NPstereo**, translates an NP structure written as absolute SMILES into the corresponding isomeric SMILES notation containing stereochemical information, with 80.2% per-stereocenter accuracy for full assignments and 85.9% per-stereocenter accuracy for partial assignments, across various NP classes including secondary metabolites such as alkaloids, polyketides, lipids and terpenes. **NPstereo** might be useful to assign or correct the stereochemistry of newly discovered NPs.

**Graphical Abstract:**

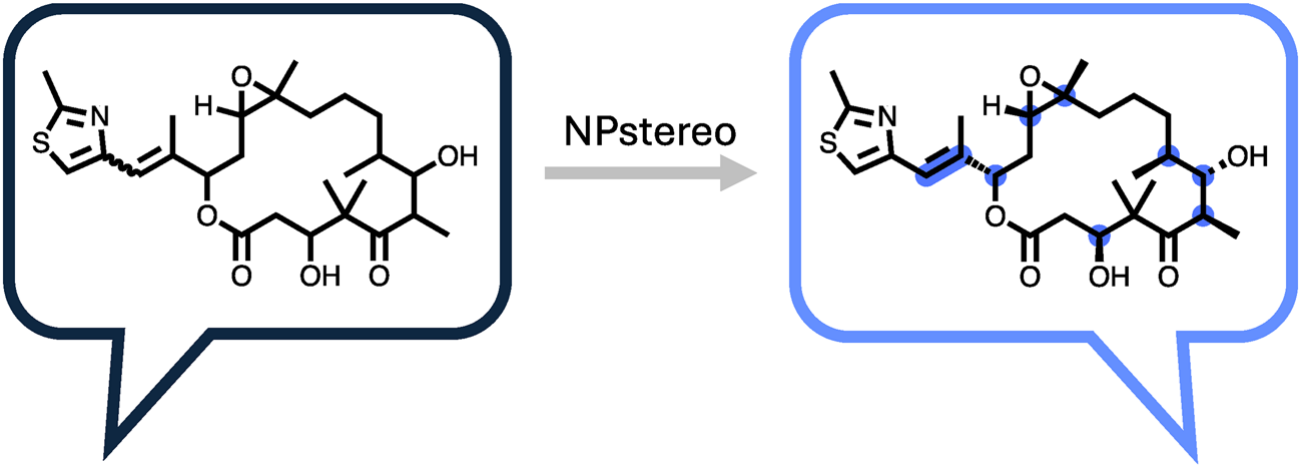

## Scientific contribution

Our study reports that a language model, NPstereo, can learn and predict the stereochemistry of natural products (NPs) from their 2D structures with high accuracy. This work demonstrates that NP stereochemical patterns are machine learnable from data and represents a first step for scalable computational methodologies for stereochemical assignment of newly discovered NPs.

## Introduction

Since the identification of carbon containing molecules as signature constituent of living matter on our planet (*vis vitalis*), deciphering the structure and function of natural products (NPs) has guided the development of organic chemistry and remains an essential source of inspiration for the development of new medicines [1–12]. However, while the atom connectivity of NPs can be assigned by a variety of methods ranging from degradation chemistry to Mass Spectrometry and NMR spectroscopy, as of today determining the configuration of individual stereocenters in NPs (3D-structure) remains challenging and requires techniques such as X-ray crystallography and chiroptical spectroscopy often combined with derivatization [13–18], and sometimes total synthesis to confirm or correct the initial stereochemical assignment [19–27].

Nature has settled for only L-chirality in proteinogenic amino acids and D-chirality in the carbohydrate backbone of nucleotides. Furthermore, conserved stereochemical patterns exist in certain natural products that derive from preserved biosynthetic pathways in organisms of common taxonomic origin [28, 29]. Here, we asked the question whether such regularities in NP stereochemistry might be sufficiently prevalent to allow this information to be machine learned. To the best of our knowledge, this question has not been addressed despite many studies using machine learning to classify NPs and their relation to other molecular classes [30–37]. We set out to test if a transformer model, a type of neural network initially developed for language translation [38], found to perform well for a variety of chemistry related tasks [39], including the prediction of stereoselective reactions in forward and retrosynthesis direction [40–42], might be able to learn NP stereochemical assignments.

As detailed below, we found that the stereochemistry of NPs can indeed be predicted by a transformer model trained on inserting missing stereochemical labels for chiral centers and *Z/E* double bonds into a SMILES (Simplified Molecular Input Line Entry System) [43, 44] string representation of the molecular structure with entirely missing or partially assigned stereocenters. We trained our model using 63,998 NPs with fully assigned stereochemistry and a literature reference, which we collected from the open access database COCONUT (COlleCtion of Open Natural ProdUcTs), a database which combines several NP databases into a single source [45]. Our transformer model, called **NPstereo**, predicts NP stereochemistry with 80.2% per-stereocenter accuracy for full assignments and 85.9% per-stereocenter accuracy for partial assignment across various NP classes.

## Results and discussion

### Dataset analysis

All NPs associated with at least one literature reference were extracted from the COCONUT database, which provided 116,403 NPs written as canonical isomeric SMILES, including stereochemical information for tetrahedral centers (@ or @@ in 68:32 ratio) and stereogenic double bonds (/C=C/ and \C=C\ for *Z*, /C=C\ and \C=C/ for *E* in 68:32 ratio). Note that while double bond *Z* vs. *E* stereochemistry directly corresponds to the possible labels, the Ingold-Prelog *R* vs. *S* do not correspond directly to @ vs. @@ labels but require considering these labels together with the order of substituents as written in the SMILES [43, 44]. Indeed the ratio of *R* to *S* chiral centers in COCONUT is 49:51. Overall, the selected dataset of 116,403 references NPs contained 550,610 annotated tetrahedral stereogenic centers and 55,296 stereogenic double bonds.

**Fig. 1:**
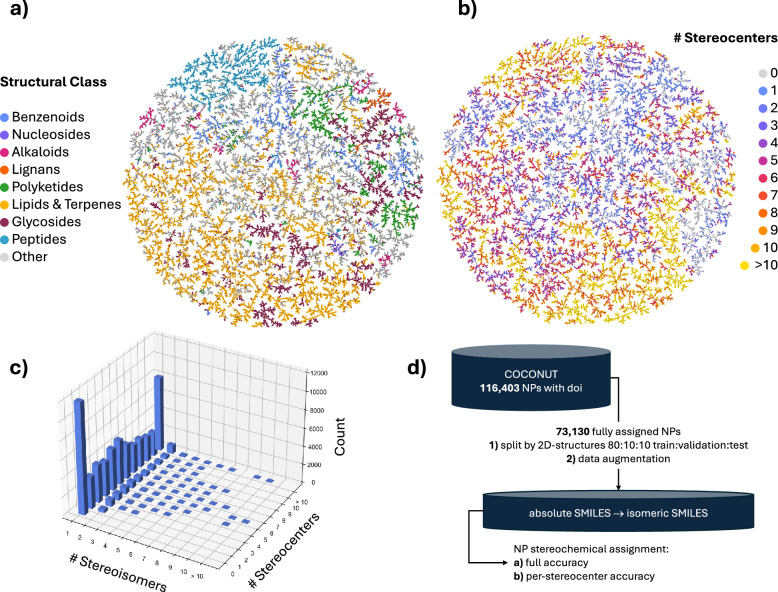
Dataset analysis and model training strategy. **a**, **b** MAP4C TMAPs of 116,403 unique compounds (canonical isomeric SMILES) with an associated DOI, extracted from the COCONUT database. The TMAP visualizations are colored according to **a** NP structural classes, as defined in the COCONUT database “chemical_super_class” field, with peptides and glycosides further refined using SMARTS substructure searches; and **b** the number of stereocenters in each structure. An interactive version of the TMAP plot can be accessed at: https://tm.gdb.tools/map4/NPstereo/. **c** 3D bar plot showing the number of molecules (vertical axis) as a function of the number of total stereocenters (depth axis) and the number of stereoisomers (horizontal axis) for 63,988 NPs with fully assigned stereocenters. **d** Workflow for data selection, model training and evaluation

Visualizing similarity relationships between NPs as measured by the MAP4C molecular fingerprint [46], using the dimensionality reduction method TMAP [47, 48], provided a layout illustrating the different structural classes (polyketides, benzenoids, nucleosides, alkaloids, lignans, peptides, lipids & terpenes, and glycosides, Fig. 1a). The structural classes differed by the number of stereocenters per molecule, which was relatively high for lipids & terpenes, peptides, and glycosides, and much lower for benzenoids and nucleosides (Fig. 1b). Some of the 116,403 NPs corresponded to different stereoisomeric forms of the same 2D-structure, including structures with incomplete of fully missing stereochemical assignment, such that the set only contained 98,108 different 2D-structures without stereochemical assignment, written as absolute SMILES. Note that NPs with incomplete stereochemical assignments were evenly distributed among the different NP structural classes.

The subset of NPs with fully assigned stereochemistry featured 73,130 different isomeric SMILES corresponding to 63,998 different absolute SMILES after removal of stereochemical labels. Among these, 12,095 absolute SMILES (18.9%) did not contain any stereocenter, 9254 (14.5%) contained 11 or more stereocenters, and the remaining 42,649 (66.6%) were approximately evenly distributed in groups containing between one and ten stereocenters. Most of these absolute SMILES corresponded to a single stereoisomer database entry (i.e. a single isomeric SMILES, 56,676, 88.6%), a small group to two stereoisomeric database entries (6100, 9.5%), and a very small fraction (1212, 1.9%) to three or more stereoisomeric database entries (Fig. 1c).

### Model design and training

To test if NP stereochemical assignment could be machine learned, we set out to train a transformer model to translate a source absolute SMILES, describing the unassigned 2D-structure of an NP, into the corresponding target isomeric SMILES containing stereochemical labels. For model training, we used the 73,130 NPs with literature reference and fully assigned stereochemistry extracted from COCONUT. We split the data into training, validation and test sets at the level of 2D-structures (63,988 canonical absolute SMILES, lacking stereochemical labels) with an 80:10:10 ratio, and optionally considered data augmentation schemes compensating for the relatively small dataset size to train and evaluate various models in terms of full assignment accuracy and per-stereocenter assignment accuracy in a three-fold cross-validation (Fig. 1d).

To train our first transformer model **C1**, we considered each canonical isomeric SMILES in the training and validation splits and generated a corresponding absolute SMILES by removing all stereochemical labels, which resulted in an absolute SMILES with the same order of characters as the canonical isomeric SMILES. These absolute SMILES were used as source strings and associated with the corresponding canonical isomeric SMILES as target strings, resulting in training and validation datasets for model **C1** (see methods for details). In this manner, model **C1** would be trained to convert each absolute SMILES into the corresponding canonical isomeric SMILES by inserting stereochemical labels without having to alter the order of characters in the SMILES.

Next, we enlarged the training and validation sets of **C1** using two possible data augmentation approaches. First, we used SMILES randomization [49], a technique which generates a number of non-canonical forms of a canonical SMILES and is often used to regularize and enhance the performance of language models trained on SMILES [50, 51]. We applied the procedure to the target lists of canonical isomeric SMILES at 2-, 5-, 10-, 20-, and 50-fold levels, and subsequently removed stereochemical labels from the resulting non-canonical isomeric SMILES to produce the corresponding absolute SMILES for the augmented source lists, which resulted in augmented datasets to train models **A2**, **A5**, **A10**, **A20** and **A50**. Second, we randomly removed stereochemical labels from each canonical isomeric SMILES of the target list in **C1** in up to five different versions for each number of removed label to produce an augmented source list of partially assigned isomeric SMILES. We then paired each of these partially assigned isomeric SMILES with their parent fully assigned canonical isomeric SMILES in the target list. This procedure resulted in a 25-fold augmentation of the data to train model **NPstereo**. Finally, we combined both data augmentation approaches by applying a tenfold SMILES randomization to the target lists of canonical isomeric SMILES of **C1**, and then randomly removing stereochemical labels from each randomized isomeric SMILES one at a time until none remained to produce partially assigned randomized SMILES for the source list. This procedure augmented the **C1** training and validation datasets by approximately 65-fold, resulting in training data for model **M65**.

In addition to these augmented datasets, we generated a negative control dataset **R1** by randomizing stereochemical labels in the target lists of canonical isomeric SMILES of model **C1** such that no pattern in stereochemistry should be recognizable. An additional negative control dataset **RP** was created by partial removal of stereochemical label from the target list of **R1** to augment the source list. All models described above were trained for approximately 9 h to complete 100,000 training steps (see methods for details).

### Performance evaluation

Transformer models produce a list of possible output when prompted with an input query. To evaluate the performance of NP stereochemistry assignment, we restricted our analysis to the top-1 output because, for NPs with a single stereocenter, the top-2 list would exhaust the list of possibilities and therefore always be correct. Top-1 performance should be evaluated compared to chance assignment, which is 50% per stereocenter and decreases exponentially with an increasing number of stereocenters (25% for 2 stereocenter, 12.5% for 3 stereocenters, etc.), a performance to be expected for our negative control models **R1** and **RP** trained with randomized stereochemical labels. Note that when challenged with an input NP absolute SMILES, our transformers must output not only the stereochemical assignment, but rather the complete NP isomeric SMILES token per token, and avoid assigning stereochemical labels when none are present, such as in NPs lacking stereocenters.

We first tested the different models on writing fully assigned canonical isomeric SMILES from canonical absolute SMILES, a task which corresponds to assigning the configuration of all stereocenters in an NP 2D-structure (Table 1, upper part, center: canonical full assignment test set). All models produced almost exclusively (> 99%) valid canonical SMILES, indicating reliable learning of the canonical SMILES syntax. In contrast, the non-canonical SMILES produced by the models were overall less valid (~ 80%). In terms of prediction accuracy, the best performing model was **NPstereo** trained on canonical SMILES including partially assigned sources, which achieved 58.4% top-1 accuracy for full assignment and 80.2% top-1 accuracy per assigned stereocenter. Both full-assignment and per-stereocenter accuracies provide complementary perspectives on model performance. While the full-assignment accuracy reflects the stringent case in which all stereocenters must be predicted correctly, the per-stereocenter accuracy captures the model’s ability to assign individual stereocenters, a situation often encountered in practice when only part of a molecule’s stereochemistry is unknown. The second-best model was **A50** with 56% top-1 accuracy for full assignment and 80% top-1 accuracy per assigned stereocenter. All other models performed worse but still significantly above the negative control models **R1** and **RP** trained with randomized stereochemical labels, which achieved ~ 24% top-1 accuracy for full assignment. This performance level reflected their ability to identify NPs lacking stereocenters (19% of the dataset), combined with the probability that a random stereochemical assignment can be correct (50% for the 6% NPs containing a single stereocenter). The inability of both negative control models to recognize stereochemical patterns was well reflected in their ~ 50% top-1 per-stereocenter accuracy, close to the random guess expectation.

**Table 1:** SMILES validity and performance metrics of models for NP stereochemistry assignment evaluated in a threefold cross validation across different dataset augmentation strategies

	Training dataset (Train + Validation)		Canonical full assignment test set^a)^			Non-canonical full assignment test set^b)^		
Model	SMILES type	Size	SMILES validity^c)^	Full-assignment accuracy^d)^	Per-Stereocenter accuracy^e)^	SMILES validity^c)^	Full-assignment accuracy^d)^	Per-Stereocenter accuracy^e)^
**C1**	Absolute → Canonical	58,571	99.8 ± 0.04	56.8 ± 0.17	79.5 ± 0.13	81.5 ± 0.6	22.8 ± 0.81	39.8 ± 1.46
**A2**	Absolute → Randomized (2x)	116,872	99.8 ± 0.03	36.7 ± 0.38	67.9 ± 0.85	80 ± 0.42	45.7 ± 0.97	72.9 ± 0.45
**A5**	(5x)	288,472	99.8 ± 0.07	43.9 ± 0.76	73.3 ± 0.35	80 ± 0.4	51.3 ± 0.55	77.1 ± 0.45
**A10**	(10x)	570,898	99.8 ± 0.03	51.5 ± 0.81	77.9 ± 0.3	80 ± 0.45	54.1 ± 0.37	79 ± 0.19
**A20**	(20x)	1,117,782	99.8 ± 0.04	54.4 ± 0.21	79.3 ± 0.15	80.1 ± 0.38	55.1 ± 0.81	79.5 ± 0.62
**A50**	(50x)	2,651,393	99.9 ± 0.03	56 ± 0.29	80 ± 0.16	80 ± 0.41	**55.4 ± 0.45**	**79.5 ± 0.34**
**NPstereo**	Partially Assigned → Canonical	1,370,809	99.9 ± 0.02	**58.4 ± 0.66**	**80.2 ± 0.37**	81 ± 0.24	23.3 ± 0.59	44.6 ± 1.67
**M65**	Partially Assigned → Canonical or Randomized	3,872,215	99.9 ± 0.03	46.1 ± 0.5	74.9 ± 0.18	80 ± 0.4	47.6 ± 0.42	74.9 ± 0.56
**R1**	Absolute → Canonical with randomized stereochemistry	58,571	99.9 ± 0.03	24.4 ± 0.65	51.3 ± 1.32	80.8 ± 0.29	19.7 ± 0.53	35.8 ± 0.82
**RP**	Partially Assigned → Canonical with randomized stereochemistry	1,757,098	99.9 ± 0.01	24.1 ± 0.83	53.3 ± 0.48	80.5 ± 0.65	19.4 ± 0.18	37.6 ± 1.22
			Canonical partial assignment test set^f)^			Non-canonical partial assignment test set^g)^		
**NPstereo**	Partially Assigned → Canonical	1,370,809	99.9 ± 0.02	**63.6 ± 0.31**	**85.9 ± 0.1**	81 ± 0.24	14.9 ± 0.6	45.9 ± 1.37
**M65**	Partially Assigned → Canonical or Randomized	3,872,215	99.9 ± 0.03	51.1 ± 0.3	81 ± 0.43	80 ± 0.4	**53.6 ± 0.56**	**81.6 ± 0.54**
**RP**	Partially Assigned → Canonical with randomized stereochemistry	1,757,098	99.9 ± 0.01	14.2 ± 0.18	51.5 ± 0.4	80.5 ± 0.65	8 ± 0.62	35.4 ± 1.24

^a)^Test set consisting of absolute SMILES generated by removing all stereochemical labels from canonical isomeric SMILES

^b)^Test set consisting of absolute SMILES generated by removing all stereochemical labels from randomized isomeric SMILES

^c)^Percentage of valid SMILES generated by the model considering the top-3 outputs

^d)^Full-assignment accuracy represents the percentage of times the isomeric SMILES (canonical or non-canonical) of the target is produced by the model in the top-1output in response to the source absolute or partially assigned isomeric SMILES (canonical or non-canonical)

^e)^The per-stereocenter accuracy is the percentage of correctly predicted stereocenters per molecule. For NPs without stereocenter, the predicted SMILES is considered correct if it matches the target SMILES. The best top-1 value for each accuracy metric is highlighted in bold. See text and methods for details

^f)^Test set consisting of isomeric SMILES generating by removing part of the stereochemical labels from canonical isomeric SMILES

^g)^Test set generated by removing part of the stereochemical labels from randomized isomeric SMILES

When tested on non-canonical absolute SMILES, **NPstereo** performed almost the same level as the negative control models **R1** and **RP** for full assignment and per-stereocenter accuracy (Table 1, upper part, right, non-canonical full assignment test set). This low performance indicated that **NPstereo**, which was trained with canonical SMILES, needed the canonical order of SMILES characters in the source to produce valid SMILES annotated with the correct NP stereochemistry. On the other hand, models **A2**-**A50** trained with both canonical and non-canonical SMILES performed similarly well on both top-1 accuracy for full assignment (45.7–55.4%), and top-1 per-stereocenter accuracy (72.9–79.5%). The mixed model **M65** trained with canonical and non-canonical partially assigned source SMILES, performed similarly to model **A5** in terms of top-1 accuracy for full assignment (46.1%) and per-stereocenter top-1 accuracy (74.9%), indicating that the addition of partially assigned SMILES was not helpful for learning the stereochemistry of NP written in non-canonical SMILES format.

We further tested models **NPstereo**, **M65** and the negative control **RP**, trained on translating partially assigned isomeric SMILES to the corresponding fully assigned SMILES, on adding missing stereochemical labels to partially assigned SMILES. This task is comparable to completing the stereochemical assignment of a partially assigned NP structure, which is often encountered in practice. When tested with partially assigned canonical SMILES, **NPstereo** was better than **M65** and performed slightly better than on the full assignment task in terms of top-1 overall accuracy (63.6%) and top-1 per-stereocenter accuracy (85.9%, Table 1, lower part, center, canonical partial assignment test set). However, the model collapsed to the level of the negative control on all three measures when tested with non-canonical SMILES, indicating again the requirement for a canonical order of characters in the source SMILES for proper prediction. On the other hand, model **M65** performed quite well for assigning missing stereocenters to partially assigned non-canonical SMILES in terms of top-1 overall accuracy (53.6%) and top-1 per-stereocenter accuracy (81.6%, Table 1, lower part, right, non-canonical partial assignment test set).

**Fig. 2:**
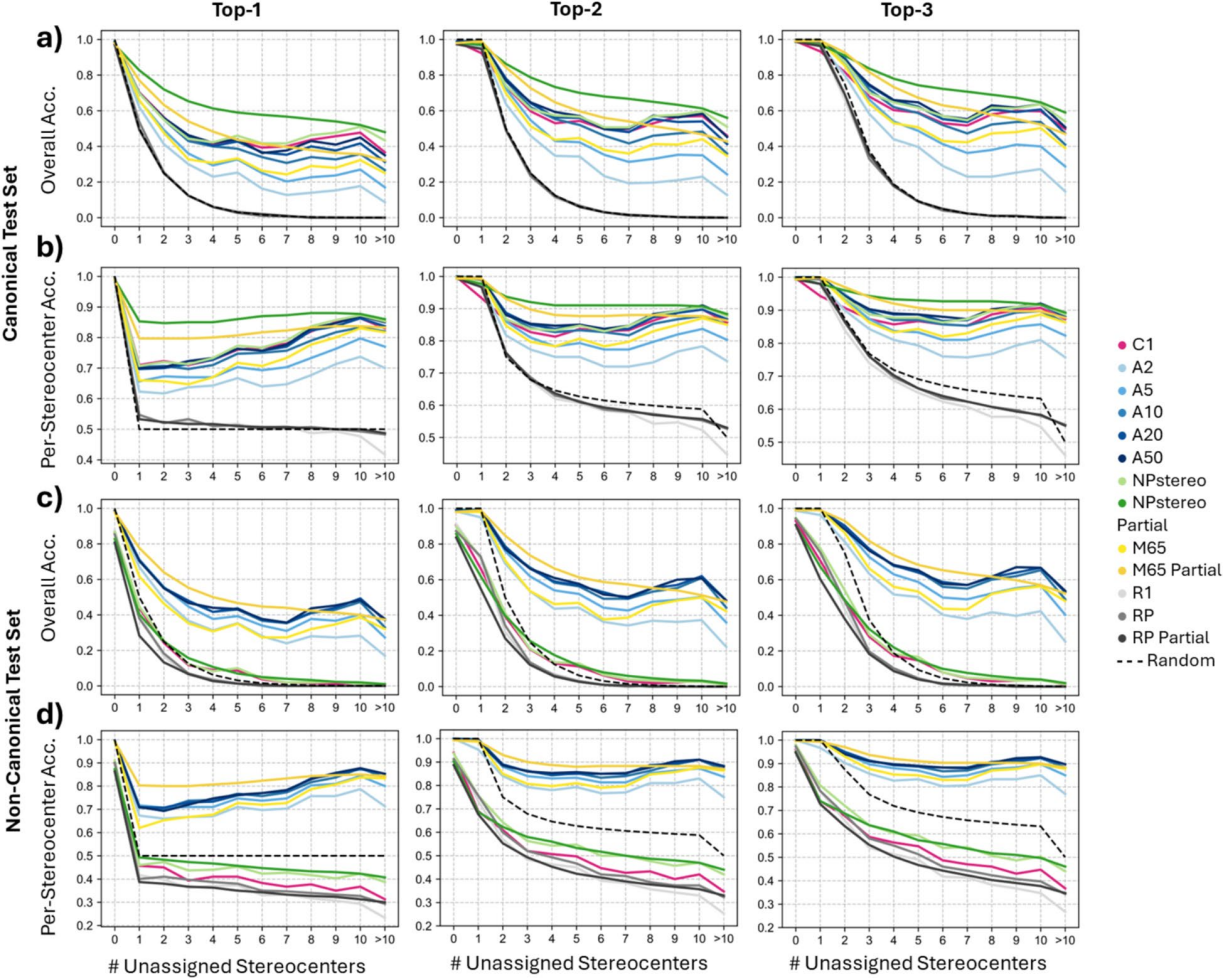
Model performance stratified by the number of unassigned stereocenters. Canonical test set top-1, top-2 and top-3 full assignment (**a**) and per stereocenter accuracy (**b**). Non-canonical test set top-1, top-2 and top-3 full assignment (**c**) and per stereocenter accuracy (**d**). Model predictions are produced in decreasing order of probability, the top-1 prediction being the most probable

Analyzing model performance in terms of both overall accuracy and per stereocenter accuracy as a function of the number of unassigned stereocenters per molecule provided further insights into model performance. For the canonical SMILES test set, the best models **NPstereo** and **A50** were closely matched in performance across all numbers of stereocenters per molecule, with an increase in performance when including top-2 and top-3 predictions, indicating that the model proposed meaningful alternatives down the top-N list of possible predictions, which are sorted in order of decreasing probability (light green and dark blue curves in Fig. 2a, b). However, these models were surpassed by **NPstereo** tested on the partial stereochemical assignment test set with up to nine unassigned stereocenters, indicating that, not unexpectedly, the availability of partial stereochemical information helped the model to assign the missing stereocenters (green curve in Fig. 2a, b). Note that the higher performance of all models for NPs with five or less stereocenters partly reflected the contribution of a chance assignment to be correct, as indicated by the performance curve of the negative control models **R1**, **RP** and **RP** on partial assignment task, which matched the performance expected from chance assignment (light grey, grey, black and dashed black lines, Fig. 2a, b). This analysis also showed that all models including the negative controls trained with randomized stereochemical labels performed perfectly with NPs lacking stereocenters, implying that they had learned not to add any stereochemical labels when no stereocenters were present.

For the non-canonical SMILES test set, the overall and per stereocenter assignment accuracy as function of the number of unassigned stereocenters highlighted the performance collapse of models **C1** and **NPstereo** to random and negative control levels for all numbers of stereocenters in both the full and the partial stereochemical assignment tasks, an effect also apparent in Table 1 discussed above (red and light green lines, Fig. 2c, d). In fact, models only trained on canonical SMILES (**C1**, **NPstereo**, and the negative controls **R1** and **RP**) performed even below chance levels and even partly failed to identify NPs without stereocenters for this non-canonical SMILES test set. On the other hand, model **A50** performed quite well for the full assignment and model **M65** for the partial assignment task on these non-canonical SMILES on which they had been trained, again with an increase in performance when including top-2 and top-3 predictions (dark blue and dark yellow lines, Fig. 2c, d). The dependence of model performance on the SMILES syntax (canonical or non-canonical) in training versus test sets showed that stereochemical assignment was learned according to the order of characters in the SMILES, which is very different in canonical versus non-canonical SMILES. This effect also explained the need for a relatively large training set (50-fold augmentation) for a model to learn stereochemistry in the more diverse context of randomized SMILES.

Analysis of per-stereocenter top-1 assignment accuracies as function of the number of stereocenters showed that, for the full assignment task on canonical SMILES (all models except partial assignment test set and negative controls, Fig. 2b) and non-canonical SMILES (all models trained with randomized SMILES, Fig. 2d), performance was lowest for NPs with a single chiral center, and gradually increased with additional stereocenters. These effects probably reflected the presence of regularities in NPs with high number of stereocenters such as homochirality in peptides and oligonucleotides and the limited stereochemical diversity of carbohydrates and steroids, because such regularities would be easier to learn for the different models in the full assignment task.

The same effect could explain the almost constant performance of the partial assignment task as the number of unassigned stereocenters increased for models **NPstereo** (green line in Fig. 2b) and **M65** (dark yellow line in Fig. 2b, d), because the difficulty to assign stereochemistry to NPs with a single stereocenter would be compensated by the ease of adding a single missing center to NPs with a large number of stereocenters. Indeed, analyzing model performance across different NP classes showed that both full and partial assignment accuracies were particularly high for glycosides, nucleosides, lipids & terpenes which include steroids, and peptides, with **NPstereo** standing out again as the best performing model across most of these classes (Table 2). In the case of peptide NPs in COCONUT, the dataset contained both L- and D-configured amino acids in a 74:26 ratio, which was almost preserved in the predictions of the test set (72:28).

**Table 2:** Top-1 per-stereocenter accuracy of the different models for NP stereochemistry assignment, stratified by NP structural class, in a threefold cross-validation. The best values for each NP structural class are highlighted in bold

Model	Alkaloids	Benzenoids	Glycosides	Lignans	Lipids & Terpenes	Nucleo-sides	Peptides	Poly-ketides	Other
Top-1 accuracy (%) on full assignment canonical test set									
** C1**	0.77 ± 0.04	0.69 ± 0.01	0.86 ± 0.01	0.73 ± 0.03	0.81 ± 0.01	0.89 ± 0.04	0.81 ± 0.01	0.75 ± 0.02	0.73 ± 0.01
** A2**	0.58 ± 0.04	0.6 ± 0.03	0.78 ± 0.02	0.63 ± 0.01	0.67 ± 0.00	0.82 ± 0.08	0.68 ± 0.03	0.67 ± 0.02	0.61 ± 0.00
** A5**	0.65 ± 0.07	0.65 ± 0.03	0.83 ± 0.00	0.71 ± 0.09	0.73 ± 0.01	0.87 ± 0.04	0.75 ± 0.00	0.71 ± 0.03	0.67 ± 0.00
** A10**	0.73 ± 0.04	0.68 ± 0.02	0.86 ± 0.01	0.7 ± 0.04	0.79 ± 0.01	0.87 ± 0.03	0.79 ± 0.02	0.76 ± 0.03	0.71 ± 0.00
** A20**	0.71 ± 0.05	0.69 ± 0.03	0.87 ± 0.00	0.73 ± 0.02	0.8 ± 0.01	0.91 ± 0.00	0.81 ± 0.01	**0.78 ± 0.02**	0.72 ± 0.01
** A50**	0.74 ± 0.03	0.7 ± 0.03	**0.88 ± 0.01**	**0.78 ± 0.02**	0.81 ± 0.00	**0.92 ± 0.05**	0.81 ± 0.01	0.77 ± 0.01	0.73 ± 0.01
** NPStereo**	**0.75 ± 0.06**	**0.71 ± 0.01**	**0.88 ± 0.01**	0.75 ± 0.05	**0.82 ± 0.01**	0.88 ± 0.05	**0.82 ± 0.01**	0.74 ± 0.01	**0.74 ± 0.01**
** M65**	0.64 ± 0.04	0.65 ± 0.01	0.86 ± 0.01	0.64 ± 0.07	0.76 ± 0.0	0.89 ± 0.07	0.78 ± 0.01	0.72 ± 0.03	0.67 ± 0.01
** R1**	0.5 ± 0.05	0.52 ± 0.01	0.46 ± 0.03	0.55 ± 0.07	0.51 ± 0.01	0.57 ± 0.03	0.38 ± 0.04	0.5 ± 0.05	0.51 ± 0.01
** RP**	0.53 ± 0.02	0.55 ± 0.04	0.5 ± 0.00	0.51 ± 0.02	0.51 ± 0.01	0.51 ± 0.06	0.49 ± 0.01	0.52 ± 0.03	0.52 ± 0.01
Top-1 accuracy (%) on partial assignment canonical test set									
** NPStereo**	**0.8 ± 0.05**	**0.76 ± 0.01**	**0.9 ± 0.01**	**0.83 ± 0.04**	**0.87 ± 0.01**	**0.91 ± 0.01**	**0.83 ± 0.01**	**0.82 ± 0.01**	**0.81 ± 0.0**
** M65**	0.73 ± 0.03	0.73 ± 0.03	0.88 ± 0.01	0.77 ± 0.04	0.82 ± 0.01	0.9 ± 0.07	0.79 ± 0.00	0.79 ± 0.01	0.74 ± 0.01
** RP**	0.54 ± 0.04	0.54 ± 0.04	0.51 ± 0.00	0.58 ± 0.04	0.51 ± 0.01	0.56 ± 0.1	0.49 ± 0.00	0.51 ± 0.01	0.53 ± 0.00

### Assigning stereochemistry with NPstereo

**NPstereo** might serve to assign the stereochemistry for partially or completely unassigned NPs. Although the inner working of transformer models is difficult to understand, one would expect the stereochemical assignments by **NPstereo** to be made in analogy to structurally similar NPs available in the training set, which would be often possible because many NPs including newly reported ones have close analogs already known and registered in the COCONUT database. In such cases **NPstereo** would most likely propose the most frequently found stereochemistry among close analogs. Such consensus assignment might not always be correct, for instance the 3:1 prevalence of L-configured amino acids should not imply that D-configuration assignments are wrong.

For our evaluation, we challenged **NPstereo** to assign stereochemistry to canonical SMILES, on which the model performed best, considering only NPs that were excluded from the training set for our model. In detail, we performed the prediction using each of the three models employed in the threefold cross-validation studies above and assigned the most frequently found stereochemistry for each stereocenter.

As anticipated, **NPstereo** consistently produced correct assignments for NPs for which close analogs were present in the training set. For instance, **NPstereo** correctly assigned the stereochemistry of tubulin binding anticancer NPs for which many analogs have been characterized and included in COCONUT, such as colchicine (**1**) [52], docetaxel (**2**) [53] and epothilone B (**3**) [54]. The peptide part of monomethyl auristatin E (MMAE, **4**), a well-known NP-derived tubulin inhibitor frequently used for antibody–drug conjugates [55], was also correctly assigned, however the first stereocenter in norephedrine attached at the* C*-terminus was misassigned to (*S*), which corresponds to the dominant configuration among the structurally close analogs of MMAE in the training set having different* C*-terminal derivatizations.

The double bond stereochemistry of the historically first isolated insect pheromone bombykol (**5**, not present in COCONUT) [56] was also correct, probably assigned by analogy to the configuration of (9*S,*10*E*,12*Z*)-hydroxy-octadecadienoic acid and related analogs present in the training data [57]. Stereochemical assignments by **NPstereo** in carbohydrates was mostly correct including the anomeric center due to the prevalence of β-D-glucosides, for example in the recently reported glucosylated plant alkaloid rhynchophylloside L 11-O-*β*-D-glucopyranoside (**6**) [58], as well as in datiscin (**7**) [59], although in this case the correct assignment of the terminal α-L-rhamnoside stereochemistry is more surprising due to the variety of 6-deoxyhexopyranoses available in NPs. It should also be noted that the full COCONUT dataset contains several datiscin stereoisomers including some represented as containing L-glucose, which are structures without references representing mostly likely erroneous entries. For the flavonoid biorobin, an isomer of datiscin, D-glucosides as well as D-galactosides have been experimentally identified [60], however non-referenced and improbable diastereomers containing L-glucose are similarly listed in COCONUT. These examples vindicate our choice of restricting the training set to referenced NPs but also highlight that correcting a stereoisomeric assignment using **NPstereo** might require expert opinion. For instance, correcting an L-glucoside to its enantiomer would be justified since L-glucose is unknown in nature, while correcting a D-galactoside to D-glucoside could be mistaken.

Further recently reported NPs such as olimycin E (**8**) [61], and mauritone A (**9**) [62], were also fully assigned correctly by **NPstereo**. On the other hand, NPstereo often misassigned stereochemistry in cases where close structural analogs had different stereochemical patterns or if no close analogs were present in the training set. Examples included misassigning a single stereocenter in the antibacterial fungal polyketide aspercitrininone A (**10**) [7], two serine amino acids (L instead of the correct D, and D instead of the correct L) and a threonine side chain (D-threonine instead of the correct D-*allo*-threonine) in the cyclic lipopeptide tetraselide (**11**), half of the stereogenic double bonds in the bacterial linear C_30_ carotenoid hydroxy-diaponeurosporenal (**12**), and three stereocenters in ( +)-nectamazin A (**13**) whose stereochemistry was recently reassigned and for which close analogs display diverse stereochemical arrangements [63]. The difficulty to assign double bond stereochemistry, as exemplified with carotenoid **12**, might partially reflect the much smaller training data available since only 10% of the stereochemical labels in our data are *Z/E* double bonds.

**Fig. 3:**
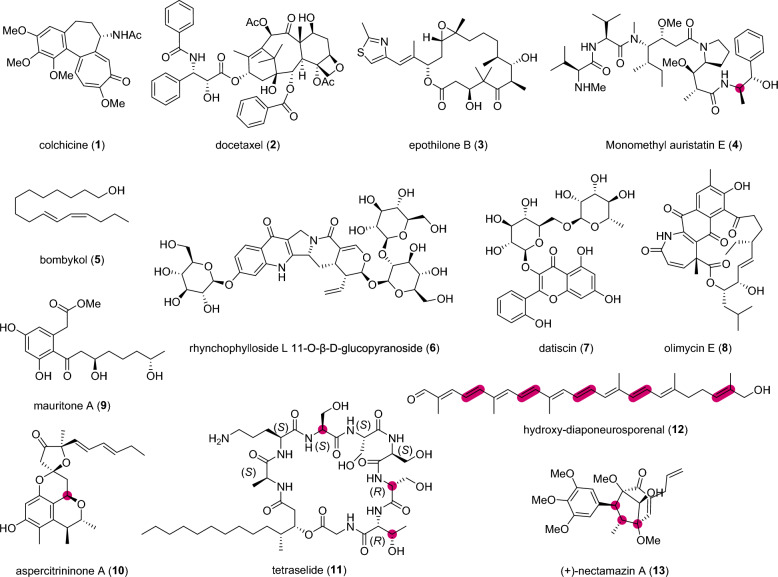
Assigning NP stereochemistry using **NPstereo**. The structural formulae of selected NPs are shown with correct stereochemistry. **NPstereo** made correct assignments in all cases except for stereocenters highlighted in red, for which it made the opposite assignment

The above examples illustrate that **NPstereo’s** predictive accuracy was strongly influenced by the local structural patterns present in the training data. Accurate predictions were obtained when high-similarity analogs with consistent stereochemistry were available. On the other hand, performance declined when such contextual information was absent (Fig. 3).

## Conclusions

In this study, we demonstrated the efficacy of transformer-based models for assigning the stereochemistry of NPs from their absolute SMILES representations using data extracted from the COCONUT database. The selected model, named **NPstereo**, was trained and challenged with canonical SMILES, and achieved a per-stereocenter accuracy of 80.2% top-1 per-stereocenter accuracy for full stereochemical assignments and 85.9% top-1 per-stereocenter accuracy for partial stereochemical assignments, with increased performance when extending beam size to top-2 and top-3 predictions, which is particularly meaningful for NPs with many stereocenters. The model showed a consistent ability to assign stereochemistry across molecules of varying complexity and performed particularly well for NPs with multiple stereocenters. In downstream evaluation on representative examples, **NPstereo** reliably predicted stereochemistry when the query NP had closely related analogs in the training set with consistent stereochemical annotations. Conversely, performance decreased for queries with low similarity to the training data or with inconsistent stereochemistry among related NPs. These findings confirm that the model leverages local stereochemical patterns and emphasize the importance of training set coverage. Overall, our work demonstrates that learnable stereochemical patterns exist in many NP classes and introduces a scalable methodology for assigning the stereochemistry of NPs, with potential to improve the method further as training sets grow in coverage and consistency. In terms of possible practical application, **NPstereo** might serve to propose possible assignments to unassigned or partially assigned NPs, and to identify potential misassignments in existing NP datasets, however taking into account the intrinsic limitations of the approach. **NPstereo** may also help identify recurrent stereochemical motifs across natural products. Such patterns, including those that deviate from canonical biosynthetic expectations, could provide hints about structural constraints or enzyme selectivity and offer insights into the broader logic underlying natural product stereochemistry. Beyond chemical structure, integrating taxonomic or genomic information into predictive models could further improve stereochemical assignment, particularly for compound classes with multiple known configurations. This represents a promising avenue for future development of **NPstereo**.

## Methods

### Dataset processing and visualization

The complete COCONUT database (09-2024) was retrieved from the website https://coconut.naturalproducts.net via the dedicated “Download” section as a PostreSQL dump. A SQL query was executed on the database to extract NPs with at least one associated citation DOI. The query retrieved the molecule identifier, canonical isomeric SMILES [43] structure and chemical class, for a total of 116,403 entries. The results were then exported as a CSV file for further processing.

The complete dataset of 116,403 NPs, consisting of isomeric SMILES with either fully or partially assigned stereocenters, were encoded using the MAP4C fingerprint [46]. The indices obtained from the MAP4C calculation were used to create a locality-sensitive hashing (LSH) forest of 32 trees. For each NP, the 20 approximate nearest neighbors in the MAP4C feature space were extracted from the LSH forest and used to calculate the TMAP layout [47, 48]. The resulting layout was displayed in a static TMAP plot using the Python matplotlib package (3.5.3). The NPs in the TMAP were color-coded to highlight structural class, stereochemistry, and dataset split. Structural class information was extracted from the COCONUT “chemical_super_class” entry and further refined for peptides and glycosides using SMARTS patterns. The number of stereocenters was calculated using RDKit (2023.9.5).

### Training data

NPs with incomplete stereochemistry (including both tetrahedral and double bond stereochemistry) were removed, reducing the dataset to 73,130 structures. These entries were then grouped by their canonical absolute SMILES (notation without stereochemistry), yielding 63,988 unique structures, each potentially associated with one or more stereoisomers. The canonical absolute SMILES dataset was divided into training, test, and validation sets using an 80:10:10 random split. In each of these three sets, each canonical absolute SMILES was associated with one or more, each written as a canonical isomeric SMILES. These canonical isomeric SMILES were used to form the target lists for training model **C1**.

**Fig. 4:**
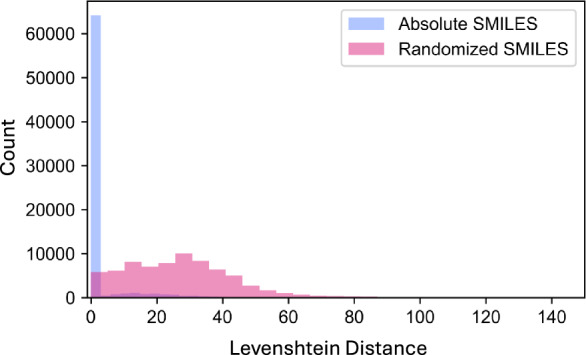
Levenshtein distance distribution comparing NP absolute canonical SMILES generated with RDKit to two alternatives: (1) the same absolute canonical SMILES with stereochemistry-defining characters removed (blue) and (2) a randomized version of the same absolute SMILES generated with RDKit (red)

To generate the corresponding source lists, we removed the stereochemical labels in each canonical isomeric SMILES to obtain an equivalent absolute SMILES. Importantly, different stereoisomers of the same canonical isomeric SMILES produced the same absolute SMILES after removing stereochemical labels, implying that the order of characters in the canonical isomeric SMILES did not contain stereochemical information. Also note that the absolute SMILES generated by removing stereochemical labels from canonical isomeric SMILES were almost identical to the canonical absolute SMILES, as measured by the Levenshtein distance between the two characters strings, compared to the Levenshtein distance between different absolute SMILES generated by SMILES randomization (Fig. 4).

To obtain additional training data, we used several data augmentation schemes in the training and validation sets separately. First, we applied SMILES randomization [49] to the canonical isomeric SMILES in the target lists of **C1** to increase their number by approximately factors of 2, 5, 10, 20, and 50 (after removal of duplicates), producing augmented target isomeric SMILES lists. We then generated the absolute SMILESs of each randomized isomeric SMILES by removing stereochemical labels for the source lists, resulting in training datasets for models **A2**, **A5**, **A10**, **A20** and **A50**. As a second data augmentation approach, we augmented the source lists of **C1** with SMILES containing only partially assigned stereochemistry. To do so, we identified the number of stereocenters (n) in each molecule. For each molecule, we created up to 5 SMILES variations by randomly removing stereochemical labels for each level of stereochemistry removal, starting with removing all stereocenters (n), then n-1, and continuing until no stereocenters were replaced. This resulted in augmented source lists of isomeric SMILES strings with progressively reduced stereochemistry, each associated with the corresponding fully assigned canonical isomeric SMILES in the target lists, composing the training data for model **NPstereo**. In a third approach, we combined augmentation through SMILES randomization with augmentation by partial removal of stereochemical labels. To do so, we first augmented the target lists of **C1** tenfold using SMILES randomization. In a second step, we augmented the source list by generating one additional variation for each level of stereochemistry removal for each randomized isomeric SMILES. This combined procedure resulted in approximately 65-fold data augmentation, providing training data for model **M65**.

Finally, we generated a first negative control training dataset **R1** by randomizing the stereochemical information in the target list of canonical isomeric SMILES of model **C1**, and a second negative control training dataset **RP** by augmenting the source lists of control **R1** using the partial assignment procedure used for model **NPstereo**.

For all datasets, isomeric SMILES (target) and the absolute SMILES generated from them (source) were tokenized using a custom tokenizer which applies a regular expression to split the SMILES string into individual chemical symbols, atoms, and bond types. The tokenizer captures elements like atoms (e.g., “Br”, “Cl”), bond types (e.g., “ = ”, “#”), and stereochemistry markers. All resulting training, validation, and test splits were saved as separate text files.

### Transformer training

Model training was carried out on the OpenNMT python ecosystem (3.5.1). [64] All models used a transformer-based architecture with 6 layers each for both the encoder and decoder, employing 8 attention heads and a hidden size of 512. Training utilized mixed precision (fp16) and Adam optimizer with a scheduled learning rate initialized at 2 and Noam decay. Batches were processed with a bucket size of 262,144 tokens and a batch size of 4096 tokens. Dropout regularization of 0.1 was applied during training, including attention dropout. The models were trained for a total of 100,000 steps. Checkpoints were saved every 25,000 steps, with validation performed every 5,000 steps to monitor model performance. The model hyperparameters and training parameters were configured according to the recommendations provided by OpenNMT, which are generally well suited for SMILES translation tasks. Complete configuration files for setup and training are available at https://github.com/reymond-group/NPstereo. Each model required approximately 9 h to complete 100,000 training steps on a single Nvidia GeForce RTX 3070 GPU. The model checkpoint at step 100,000 was selected for subsequent performance evaluation across all trained models.

### Performance evaluation

All calculations were done using the NumPy (1.26.4), pandas (2.1.0), and RDKit (2023.9.5) python libraries. All models were evaluated in a threefold cross-validation. The following performance metrics were used:

*SMILES validity* Ratio of valid SMILES to the total number of predicted SMILES.

*Full-assignment accuracy* Ratio of correctly predicted isomeric SMILES strings to the total number of predicted isomeric SMILES. An isomeric SMILES string predicted from an absolute SMILES is considered correctly predicted if it matches exactly one of the isomeric SMILES associated with this absolute SMILES.

*Per-stereocenter accuracy* Average ratio of correctly predicted stereocenters within a single prediction, accounting for both tetrahedral stereocenters and stereogenic double bonds. When multiple associated stereoisomers are present, the highest ratio is used.

### Inference

For predictions on new natural products, all three models trained in the cross-validation are used to predict the stereochemistry assignment. The final prediction is obtained via majority scoring, which consists of taking the label that was predicted the most for every stereocenter.

## Data Availability

The code for data extraction and augmentation, training the transformer models, running predictions, and analyzing results is available at https://github.com/reymond-group/NPstereo. The datasets used to train the models can be downloaded from https://zenodo.org/records/13790363.
